# Cross-Linked Polythiomethacrylate Esters Based on Naphthalene—Synthesis, Properties and Reprocessing

**DOI:** 10.3390/ma13133021

**Published:** 2020-07-06

**Authors:** Karolina Fila, Beata Podkościelna, Maciej Podgórski

**Affiliations:** 1 Department of Polymer Chemistry, Institute of Chemical Sciences, Faculty of Chemistry, Maria Curie-Sklodowska University, Maria Curie-Sklodowska Sq. 5, 20-031 Lublin, Poland; beatapod@poczta.umcs.lublin.pl (B.P.); maciej.podgorski@colorado.edu (M.P.); 2 Department of Chemical and Biological Engineering, University of Colorado, UCB 596, Boulder, CO 80303, USA

**Keywords:** cross-linked polymers, methyl methacrylate, thermal properties, thiol-thioester exchange, thiol-ene chemistry, recycling polymers

## Abstract

Two structurally different aromatic dithioesters were synthesized from two dithiols and methacryloyl chloride. The polymer networks based on methyl methacrylate and/or styrene and the new dimethacrylates were subsequently prepared. The polymerization yields of copolymers were in the range of 95–99%. The thermal and mechanical properties of the copolymers were determined by means of differential scanning calorimetry (DSC), thermogravimetric analysis (TG/DTG), and Shore D hardness. The addition of dithioesters—1,5-NAF-S-Met (or 1,4(1,5)-NAF-CH_2_S-Met) (from 0.5% to 5%) to MMA- or ST-based polymers results in lowering the glass transition temperature (T_g_) by about 8 °C. The thioester-containing polymers based on MMA exhibit lower thermal stability than those with ST. The polythioesters are stable up to 250 °C. The UV/vis spectra and refractive indexes of prepared liquid compositions were also measured. The 1,5-NAF-S-Met (and 1,4(1,5)-NAF-CH_2_S-Met) improved the refractive index values of ST and MMA compositions. The double bond conversion was also determined for all synthesized materials. The swelling studies of polymers with 20% addition of thioester crosslinkers were investigated. For all polymeric materials with 20% addition of thioesters, depolymerization of the network was carried out by thiol-thioester exchange. The depolymerization products were re-reacted in a thiol-ene reaction with 2-hydroxyethyl methacrylate by thermal initiation. The thiol-ene procedure enabled reprocessing of starting polymers and obtaining new materials characterized by distinctly different thermal, mechanical, and swelling properties. The thiol-ene materials exhibit a lower Shore hardness in the range of 20–50 °Sh, as well as decreased T_g_ values when compared to starting copolymers. Due to these possible exchange reactions, one can facilely manipulate the properties of the polymers which could lead to the manufacturing of the new products with the desired features. Degradation of the cross-linked structure and recycling of copolymers were also discussed.

## 1. Introduction

The thiol group is one of the groups classified as the most reactive functionalities in the organic chemistry [1]. It is widely known that thiols are used as efficient, nearly ideal chain transfer agents in the synthesis of common vinyl-based “optical” polymers such as methyl methacrylate (MMA) and styrene (ST) [2,3]. Lowering molecular weight for polymers, contributes to the improvement of their processing properties. High efficiency of thiols in the control of the molecular weight distribution was attributed to the weakness of the S–H bond and high reactivity of thiyl radicals which can decrease the polymer molecular weight without a significant change in the polymerization rate [4,5,6]. Only recently Encinas and co-workers [7,8] have studied, systematically, the behavior of 4-substituted thiophenols as chain transfer agents in the free-radical photopolymerization of vinyl monomers both in the aqueous solution and organic media. In another paper Krongauz and Chawla [9] investigated the influence of aromatic thiols on kinetics of acrylate radical photopolymerization in the presence and absence of photoinitiators. Moreover, the unpleasant odor of small molecular thiols limits their use in polymerization and small molecular fragments will remain in the coating layer. So far, studies on the macromolecular and grafted thiols have not been the subject of special attention.

In the field of click chemistry, there has been rapid development in many polymer related areas since Sharpless first established the characteristics of click reactions in 2001 [10]. Reactions classified as “click” show a number of features that determine their usefulness in chemical synthesis: Short reaction times; high yields; wide functional groups and tolerance to solvents; regio- and chemoselectivity; insensitivity to oxygen or water; mild, solventless (or aqueous) reaction conditions; little or no by-products; and easy purification and atoms saving up to 100% [11,12,13]. The thiol-ene reaction [12,14,15,16,17] is one of the most important click reactions because the resulting carbon-sulfur bond [18,19,20] is widely found in natural products, pharmaceuticals, bioconjugates, modern materials, polymer chemistry, and, most importantly, used for the synthesis of various drug molecules. The thiol-ene reaction can proceed either via radical addition of a thiol to a C=C ene-compound, initiated by azo- or photoinitiators subjected to heat or UV light [21,22,23,24] and also carry on via a base-catalyzed Michael addition of a thiol to an activated ene-compound with an electron deficient C=C bond, such as acrylates, maleimides, acrylamides, or derivatives thereof [25,26]. The primary amines or phosphines were used as nucleophilic bases [27].

It has been shown that dynamic covalent chemistry (DCC) is a very useful tool in the design of recyclable materials [28]. The most potent dynamic chemistries used in dynamic thermoset materials are carboxylate transesterification [29,30], olefin metathesis [31], disulfide exchange [32], Diels–Alder reaction, vinylogous urethanes [33], imine amine exchange [34], and the boronic acid-ester exchange [35]. Recently, Bowman et al. [36,37,38] systematically investigated polythioesters as a new class of covalent adaptable networks (CANs) which are subject to strong dynamic associative exchange at ambient conditions. By using base/nucleophile catalyzed thiol-thioester exchange reactions, they prepared crosslinked polythioesters because of thiol-ene photopolymerization, which are degraded in the presence of excess thiols and a catalytic amount of organic base within 3 h at room temperature. They successively studied the combination of the thiol-ene photopolymerization reaction and thiol-thioester exchange as a strategy to create a robust, recyclable, and repolymerizable materials. Hoyle et al. [22] reported a detailed review on thiol-ene reactions from recent historical and on-going industrial perspectives, as well as discussing thiol-ene reactions in detail as a newly rediscovered pathway to affording novel polymeric materials with specific properties.

Polymethacrylates like poly(methyl methacrylate) are widely used in materials with high visible light transmission [39,40]. However, their use is limited due to the high glass transition temperature. The addition of thioesters not only reduces the molecular weight of the obtained polymers but, above all, increases their processing properties.

Herein, we report an effective method for the preparation of two dithiomethacrylate monomers —1,5-NAF-S-Met and 1,4(1,5)-NAF-CH_2_S-Met, and demonstrate their novel applications in recyclable materials. This will potentially generate a high level of interest in materials science. The polymerization of these monomers with ST and MMA via a bulk polymerization technique is investigated in detail. The spectroscopic studies of the obtained thiols and thioacrylates are characterized in detail by ^1^H NMR, ^13^C NMR, and attenuated total reflectance (ATR)/FT-IR analysis. All new polymer networks with addition of thiomethacrylates were analyzed using ATR/FT-IR, Shore durometer as well as their thermal stability and transitions are determined using TGA and differential scanning calorimetry (DSC) methods. Furthermore, the optical properties were assessed. The cross-linked polymers were subjected to thiol-thioester exchange followed by thiol-ene click reactions to demonstrate reprocessability and recyclability of these materials. The novelty of the presented research stems from using aromatic dithiomethacrylates with reduced thioester bond stability. This property enables efficient thiol-thioester exchange and then in thiol-ene reactions. The thiol-thioester exchange was not yet studied in commodity MMA or ST copolymers modified with the addition of thiomethacrylate esters. The prepared materials offer new technological advancements compared to traditional esters due to the possibility of repeatable replacement of thioester bonds with the use of various commercial thiols. In turn, the presence of free thiol groups in the material allows the thiol-ene reaction to be carried out, thus, facilitating reprocessing into more environmentally friendly materials. Thiomethacrylates are expected to form stiffer materials with a higher modulus and higher T_g_ values compared to thioacrylates. Additionally, we prepared two novels bifunctional thiomethacrylates which can be of importance for high index crosslinkers for optical plastics. To enhance the high refractive index, we provided the polythioesters based on MMA or ST polymers that possess a sulfur atom having a high atomic refraction and an aromatic ring with a high molecular refraction, simultaneously. These unique properties of methacrylate thioesters can inspire new applications in materials engineering.

## 2. Materials and Methods

### 2.1. Materials in Experiments

Naphthalene, *p*-methoxyphenol, triethylbenzylammonium chloride (TEBACl), methacryloyl chloride, paraformaldehyde, ethylene chloride, chlorosulfonic acid, methylene chloride, methyl methacrylate, triethylamine (TEA), and 2-hydroxyethyl methacrylate were purchased from Sigma-Aldrich (Germany). Sulfuric acid, sodium bicarbonate, sodium chloride, tin(II) chloride hydrate, hexane, chloroform, hydrochloric acid, potassium hydroxide, sodium hydroxide, acetic acid, styrene, thiourea, ethanol, benzene, ammonium chloride, magnesium sulfate, cyclohexane, dichloromethane (DCM), methanol (MeOH), acetone, acetonitrile (ACN), and tetrahydrofuran (THF) were obtained from Avantor Performance Materials (Gliwice, Poland). α,α’-Azoiso-bis-butyronitrile (AIBN) was purchased from Merck (Darmstadt, Germany). The 4,4′-thiobisbenzenethiol (TBT) was synthesized at the Department of Polymer Chemistry (Lublin, Poland). All chemicals were used as received.

### 2.2. Analytical Methods

The Fourier transform infrared (FT-IR) spectra were recorded with a Bruker Tensor 27 FTIR spectrometer (Bruker, Ettlingen, Germany) using the attenuated total reflectance technique. The samples were thin films. All spectra were obtained at room temperature after averaging 32 scans between 600 and 4000 cm^−1^ with a resolution of 4 cm^−1^ in the absorbance mode.

The ^1^H and ^13^C NMR spectra were recorded on a Bruker Avance 300 MSL instrument (Bruker, Rheinstetten, Germany) operating at 300 MHz for ^1^H and 75 MHz for ^13^C resonance frequency. Chemical shifts were referenced to deuterated chloroform (CDCl_3_) which served as an internal standard.

The elemental analysis CHS was made using a Vario EL III analyser (Elementar Analysesysteme GmbH, Langenselbold, Germany).

Thermogravimetry (TG/DTG) was performed with a Netzsch STA 449 F1 Jupiter thermal analyzer (Netzsch, Selb, Germany) under the following operational conditions: The heating rate of 10 K min^−1^, a dynamic atmosphere of helium (flow 20 mL min^−1^), temperature range of 30–800 °C, sample mass ~10 mg, and sensor thermocouple type S TG-DSC. All TG measurements were taken in Al_2_O_3_ crucibles. As a reference, an empty Al_2_O_3_ crucible was used.

Calorimetric measurements were conducted in the Netzsch DSC 204 calorimeter (Netzsch, Selb, Germany) operating in a dynamic mode. The dynamic scans were performed at a heating rate of 10 K min^−1^ from room temperature to a maximum of 550 °C in the nitrogen atmosphere (30 mL min^−1^). The sample mass was ~10 mg. As a reference, an empty aluminum crucible was used. The reported transitions came from the first and second heating scans. Glass transition temperatures (T_g_s) of the samples were defined as the inflection point on the curves of the heat-capacity changes. The parameters such as: Decomposition temperatures (T_onset_, T_offset_), final decomposition temperature (T_d_), and enthalpy of decomposition (ΔH_d_) were also determined.

The ultraviolet-visible (UV/vis) spectra of the liquid compositions based on MMA and ST were determined by a UV-2550 (Shimadzu, Kioto, Japan) UV spectrophotometer in the range of 200–1000 nm and at a scanning rate of 200 nm/min.

Refractive index was measured at 23 °C by using Conbest Abbe’s Refractometer Type 325 (Krakow, Poland) instrument according to Method A of European Standard EN ISO 489:1999 (The Netherlands).

The Shore hardness tests were performed with a Zwick 7206/H04 durometer (ZwickRoell, Ulm, Germany), type D. The readings were taken after 15 s at the temperature of 21 °C.

The swelling coefficients (*B*) were determined from the equilibrium swelling in chosen organic solvents and distilled water, which are calculated using the Equation (1) below:

(1) $$B=\frac{m_{s}-m_{d}}{m_{d}}\times 100\%$$

where *m_s_* is the mass after swelling and *m_d_* is the mass of the dry sample.

## 3. Synthesis of Thiols

### 3.1. Synthesis of Naphthalene-1,5-Dithiol (1,5-NAF-SH)

The detailed information about syntheses of thiols (Section 3.1 and Section 3.2) are presented in Supplementary Materials (SM) S1.1–S1.2.

#### 3.1.1. Chlorosulfonation of Naphthalene [41]

Briefly, to 70 g of a naphthalene melt maintained at 80 °C in a 500 mL four-neck flask, 150 mL of chlorosulfonic acid were added from a dropping funnel during a period of 3 h, while continuously stirring. HCl gas was evolved during the resulting exothermic reaction, the bulk of which took place during the first half hour. Then the flask was placed in an ice bath and 150 mL of chlorosulfonic acid was again dropped in at 10 °C for 1 h. While warming to room temperature stirring was continued for an additional 2 h, at the end of which time precipitation occurred. The precipitate was filtered and washed twice, each time with 15 mL of chlorosulfonic acid. The resulting white solid was then slurred in 200 mL of icewater, filtered, washed with a small amount of distilled water, and finally dried, yielding 66 g of naphthalene-1,5-disulfonyl dichloride. The obtained precipitate was then purified by crystallization. Then, 50 g of a white, crystalline precipitate was obtained having a melting point of 176–178 °C (literature value 181–183 °C) [41,42]. The elemental analysis: Found for C, 37.68%; H, 1.96%; S, 19.64%; calculated for C, 36.93%; H, 1.85%; and S, 19.69%. ^1^H NMR (300 MHz, CDCl_3_, δ, ppm): 7.96–8.02 (d, 2H), 8.59–8.62 (d, 2H), and 9.27–9.30 (d, 2H); ^13^C NMR (75 MHz, CDCl_3_, δ, ppm): 127.95, 129.06, 131.06, 132.74, and 141.21 (2ArC).

#### 3.1.2. Reduction of Naphthalene-1,5-Disulfonyl Dichloride

To a flask with a capacity of 1000 mL equipped with a mechanical stirrer and thermometer were added 200 g of SnCl_2_∙H_2_O and 700 mL of CH_3_COOH. Following this, 100 g of ammonium chloride was placed in a 2000 mL conical flask, then 150 mL of sulfuric acid were added dropwise. Then the gaseous HCl was passed through the mixture which was cooled to <5 ° C until a clear solution was obtained. Next, 25 g of naphthalene-1,5-sulfonyl chloride was added to the cooled reduction liquid. Stirring was continued for 2 h. The contents of the flask were then poured into a beaker containing 1500 mL of distilled water. The formed precipitate was dissolved in an aqueous NaOH solution, and then acidified with HCl. The obtained naphthalene-1,5-dithiol was recrystallized from a mixture of CH_3_COOH and HCl. Then, 11 g (74% yield) of a light-yellow solid was obtained having a melting point of 115–118 °C (literature value 118–121 °C) [43]. The elemental analysis: Found for C, 63.29%; H, 4.92%; S, 35.36%; calculated for C, 62.5%; H, 4.17%; and S, 33.33%. ^1^H NMR (300 MHz, CDCl_3_, δ, ppm): 1.89–1.94 (m, 2H), 4.19–4.24 (-SH), 7.40–7.64 (m, 3H), and 8.05–8.17 (m, 3H); ^13^C NMR (75 MHz, CDCl_3_, δ, ppm): 124.51, 126.44, 129.56, and 133.11 (2ArC). Figure S1 (see Supplementary Materials) presents the synthesis of naphthalene-1,5-dithiol.

### 3.2. Synthesis of Naphthalene-1,4(1,5)-Di(Ylmethanethiol) (1,4(1,5)-NAF-CH_2_SH)

#### 3.2.1. Reaction of Naphthalene with Paraformaldehyde

100 g of naphthalene, 95 g of paraformaldehyde, 88 mL of glacial acetic acid, and 280 mL of concentrated hydrochloric acid were put into the 1000 mL three-necked flask. This mixture was heated in a water bath at 80–85 °C and vigorously stirred for 6 h. Then, it was transferred to a 2000 mL separatory funnel and the crude product was washed first with two 500 mL portions of distilled water, then with 500 mL of 10% sodium chloride solution, and finally with 500 mL of cold water. Figure S2A (see *SM*) presents the structural formulas of the products: 1,4 and 1,5-bis(chloromethyl)naphthalene isomers (1:1) and 1-chloromethylnaphthalene. The monosubstituted derivative is a liquid and because of filtration it is removed from the resulting mixture. The precipitate containing the mixture of isomers 1,4- and 1,5-bis(chloromethyl)naphthalene was washed with cyclohexane and then crystallization was conducted. Then, 38 g of pure solid 1,4(1,5)-bis(chloromethyl)naphthalene (yield 38%) was obtained, melting at 112–120 °C (literature value 125–130 °C) [44]. The elemental analysis: Found for C, 65.32%; H, 4.46%; calculated for C, 64.03%; and H, 4.45%. ^1^H NMR (300 MHz, CDCl_3_, δ, ppm): 5.05–5.07 (m, 8H, -CH_2_Cl), 7.50–7.69 (m, 6H), and 8.19–8.23 (m, 6H); ^13^C NMR (75 MHz, CDCl_3_, δ, ppm): 44.22–44.56 (-CH_2_Cl), 124.46, 125.52, 126.05, 127.00, 127.94, 131.52, 133.83, and 134.57 (2ArC) [44,45,46].

#### 3.2.2. Reaction of 1,4(1,5)-Bis(Chloromethyl)Naphthalene with Thiourea

In the 1000 mL round bottom flask fitted with a reflux condenser, 52 g of thiourea in 400 mL of water was dissolved and then 70 g of 1,4(1,5)-bis(chloromethyl)naphthalene and 100 mL of 96% ethanol were added. The flask was heated to 90 °C and kept under gentle reflux for 1.5 h. A solution of NaOH was prepared, poured into the flask, and heated for 1 h. After cooling the solution was filtered and 120 mL of HCl was added in portions to the filtrate. The formed precipitate was filtered off and purified by precipitation with the solution of NaOH and HCl. Next, 60 g of naphthalene-1,4(1,5)-di(ylmethanethiol) was obtained. Then crystallization with acetic acid was carried out. Then, 40 g of recrystallized naphthalene-1,4(1,5)-di(ylmethanethiol) was obtained as a light yellow solid; the melting point at 87–95 °C (literature value 80–120 °C) [47]. Figure S2B (see *SM*) shows the modification scheme for the naphthalene-1,5-di(ylmethanethiol) isomer, the reaction is analogous for the naphthalene-1,4-di(ylmethanethiol). The elemental analysis: Found for C, 66.38%; H, 5.85%; S, 30.04%; calculated for C, 65.45%; H, 5.45%; and S, 29.09%. ^1^H NMR (300 MHz, CDCl_3_, δ, ppm): 1.89–1.94 (m, 2H), 4.19–4.24 (-SH), 7.40–7.64 (m, 3H), and 8.05–8.17 (m, 3H); ^13^C NMR (75 MHz, CDCl_3_, δ, ppm): 27.14, 27.42 (-CH_2_), 124.10, 125.06, 126.77, 131.75, 137.27, and 138.29 (2ArC).

## 4. General Procedure for the Methacrylation of Thiols

The method of the preparation of aromatic thioesters was published in [48].

*S,S’-naphthalene-1,5-diyl bis(2-methyl prop-2-enethioate) (1,5-NAF-S-Met)* 7.45 g; 66% yield; solid; melting point 108–115 °C. Purification: Silica gel column chromatography (70% hexane/30% chloroform); the elemental analysis: observed for C, 64.38%; H, 4.87%; S, 18.28%; calculated for C, 65.85%; H, 4.88%; and S, 19.51%. ^1^H NMR (300 MHz, CDCl_3_, δ, ppm): 2.06 (m, 3H); 5.80–5.81 (d, 1H); 6.38 (d, 1H); and 7.57–7.62, 7.77–7.79, and 8.35–8.38 (m, 6H). ^13^C NMR (75 MHz, CDCl_3_, δ, ppm): 18.71 (–CH_3_); 124.82 (=CH_2_); 127.16, 128.72, 135.85, and 136.40 (2ArC); 143.97 (=C); and 191.23 (C=O).

*S,S’-naphthalene-1,4(1,5)-di(ylmethane) bis(2-methylprop-2-enethioate) (1,4(1,5)-NAF-CH_2_S-Met)* 7.2 g; 64% yield; liquid. Purification: Silica gel column chromatography (70% hexane/30% chloroform); the elemental analysis: observed for C, 66.16%; H, 5.46%; S, 16.96%; calculated for C, 67.42%; H, 5.62%; and S, 17.98%. ^1^H NMR (300 MHz, CDCl_3_, δ, ppm): 1.98–2.03 (m, 6H); 4.57–4.63 (m, 4H); 5.58 (d, 2H); 6.07 (d, 2H); and 7.45–7.61 and 7.88–8.05 (m, 6H). ^13^C NMR (75 MHz, CDCl_3_, δ, ppm): 18.01 (–CH_3_); 31.02–31.26 (–CH_2_S); 123.32, 124.02, and 124.51 (=CH_2_); 126.11, 126.27, 127.26, 127.51, 127.77, 131.76, 131.85, 132.56, 133.12, and 133.63 (2ArC); 143.21 (=C); and 192.60 (C=O).

**Figure 1:**
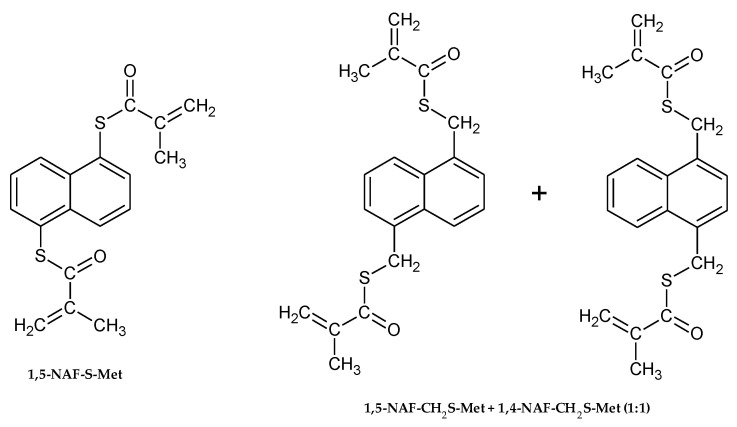
The structures of synthesized thioesters.

The chemical structures of aromatic thiomethacrylates are shown in Figure 1.

## 5. Polymerization Reactions

Bulk polymerization reactions were performed in test tubes. To each test tube containing different amounts of thioester—1,5-NAF-S-Met (0.5%, 1%, 2%, 3%, 5%, and 20% *w*/*w*), 2 g of methyl methacrylate (or styrene) was added. Then, 0.75% w/w of AIBN put into appropriate tube, mixed, and placed in a water bath for 24 h at 60 °C, and following 6h at 80 °C [48]. All obtained products were dried in a recirculating oven. To compare the results, MMA, and ST homopolymers were also made. In the same way, the copolymerization was carried out for another thioester —1,4(1,5)-NAF-CH_2_S-Met. The amounts of monomers used in copolymerization and polymerization yields were listed in Table 1.

## 6. The Thiol-Thioester Exchange

The thiol-thioester exchange was carried out on cross-linked polythioesters based on MMA and ST (1a–4a). Cross-linked polymers (with 20% addition of each dithiomethacrylate) were ground and kept in 72 h in DCM. After this time, TBT was added to each sample in a 2 molar excess relative to the introduced polythioester. Then a basic catalyst (TEA, 10% mol) was added and the resulting mixture was heated for 16 h at 60 °C. Newly obtained polymers (1b–4b) were analyzed by ATR/FT-IR spectra and DSC study. The designation of all samples prepared because of thiol-thioester exchange and thiol-ene reactions was presented in Table 2.

## 7. Thiol-ene Reactions with 2-Hydroxyethyl Methacrylate

In the next stage, the thiol-ene reaction was performed. An extensive review on thiol-ene reactions showed that the equivalence thiol:ene ratios usually range from 2:1 to 10:1 in many publications that described high reaction yields and good selectivity [49,50,51]. Furthermore, equivalence initiator:thiol ratios commonly range from 0.1 to 1 [49,50] and the most often used thermal initiator is AIBN [21,49,51,52]. Therefore, in current thiol-ene reactions, thiol materials (1b–4b) and HEMA were used in the equivalence thiol:ene proportion of 1:2, using AIBN as the initiator with the equivalence initiator:thiol ratio of 1:2.

Then, 1 g of previously prepared compound (1b), containing pendant thiol groups, 7 mL of DCM, and 0.2 g of 2-hydroxyethyl methacrylate (HEMA) was added into a round bottom flask attached to a condenser. After mixing and reaching the desired temperature, 0.05 g AIBN was fed into the flask. The reaction mixture was kept at 80 °C for 4 h under constant mechanical stirring (600 rpm). The resulting product was dried for 24 h at room temperature and then washed with acetone before ATR/FT-IR analysis. The same procedure of thiol-ene reaction was conducted for compounds 2b–4b.

**Figure 2:**
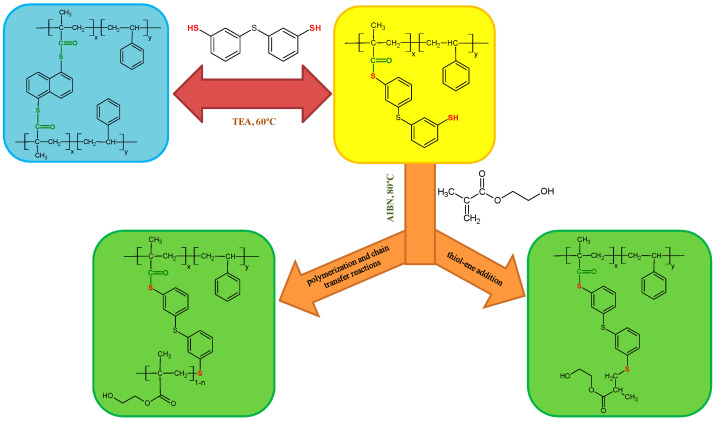
Scheme of thiol-thioester exchange and possible reaction pathways for the thiol-methacrylate system in our studies.

The exemplary scheme of the modifications carried out on the starting ST+1,5-NAF-S-Met copolymer is proposed below (Figure 2). There are two possible reaction pathways for the thiol-methacrylate system. When methacrylates are polymerized with thiol-enes, the methacrylate functional group has a strong tendency for homopolymerization and causes a pseudo two-stage polymerization [53,54,55]. The first stage is dominated by homopolymerization of methacrylate and chain transfer and the second stage is dominated by thiol-ene polymerization, as shown in Figure 2. The kinetics of the thiol-methacrylate system was investigated by Bowman et al. [53].

## 8. Results and Discussion

### 8.1. The ATR/FT-IR Spectra of Thiols and Thioesters

The comparison of ATR/FT-IR spectra before and after thioesterification of naphthalenethiols with methacryloyl chloride was presented in Figure S3 (*SM*). The most important vibrational bands present in these compounds are given below.

FTIR (cm^−1^) of the naphthalenethiols (1,5-NAF-SH and 1,4(1,5)-NAF-CH_2_SH): 3035–3048 (C–H stretching of naphthalene ring); 2940 and 2851 (asymmetric and symmetric C-H stretching vibrations); 2533–2556 (stretching vibration of –SH group); 1579–1591 (carbon-carbon stretching); 1436 (–CH_2_ deformation vibration of –CH_2_S group); 734–786 (C–H out-of-plane deformation vibrations); and633–641 (–C–S stretching).

FTIR (cm^−1^) of the naphthalene thioesters (1,5-NAF-S-Met and 1,4(1,5)-NAF-CH_2_S-Met): 3046–3084 (C–H stretching of naphthalene ring); 2926–2986 and 2844–2846 (asymmetric and symmetric C–H stretching of CH_2_ and CH_3_, respectively); 1658–1667 (stretching vibration of –C=O group); 1584–1629 (carbon-carbon stretching); 1448 (–CH_2_ deformation vibration of –CH_2_S group); 883–1025 (O=C–S valency bond); 735–789 (C–H out-of-plane deformation vibrations); and 639–664 (–C–S stretching).

### 8.2. The ATR/FT-IR Spectra of MMA and ST Copolymers

**Figure 3:**
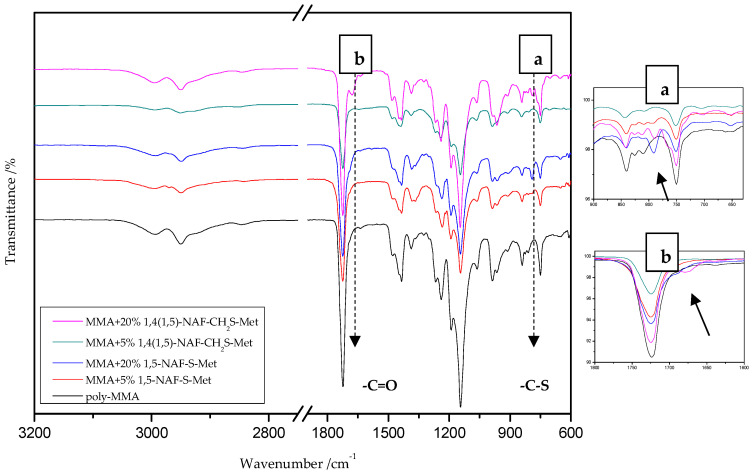
The attenuated total reflectance (ATR)/FTIR spectra of poly-methyl methacrylate (MMA) and its copolymers with 5% and 20% addition of 1,5-NAF-S-Met and 1,4(1,5)-NAF-CH2S-Met.

**Figure 4:**
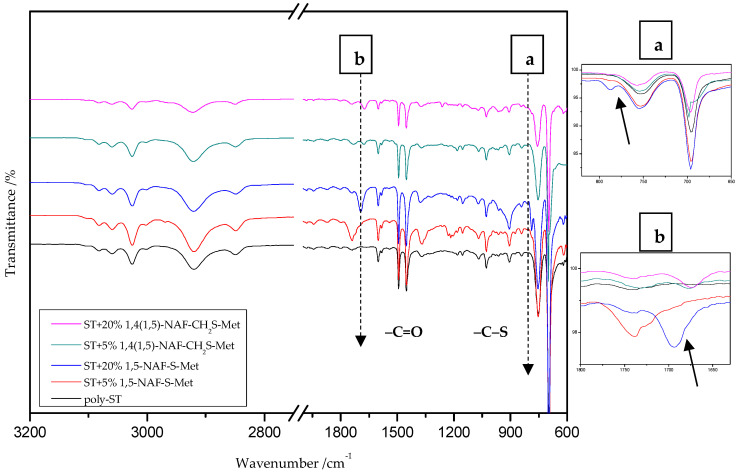
The ATR/FTIR spectra of poly-styrene (ST) and its copolymers with 5% and 20% addition of 1,5-NAF-S-Met and 1,4(1,5)-NAF-CH2S-Met.

Figure 3 and Figure 4 present the ATR/FT-IR spectra for MMA and ST copolymers containing thioesters (1,5-NAF-S-Met and 1,4(1,5)-NAF-CH_2_S-Met) in their structures. The largest changes were noticed in the spectra containing 5% and 20% addition of the synthetic thioesters, therefore these results are presented in Figure 3 and Figure 4. Data from the ATR/FT-IR analysis for all synthesized copolymers are presented in Tables S1 and S2 (see Supplementary Materials).

On the presented spectrum of MMA copolymers (Figure 3) one can see similarity in the occurring signals. For poly-MMA the bands around 2994, 2950 (asymmetric), and 2846 cm^−1^ (symmetric) are assigned to the C–H stretching vibrations. The bending vibration of the methyl (–CH_3_) group appeared at 1479 and 1435 cm^−1^ in the ATR/FT-IR spectra whereas the deformation mode of the methylene (–CH_2_–) group appeared at 1388 cm^−1^. In addition, the sharp and intense bands at 1724 and 750 cm^−1^ are attributed to the stretching and out-of-plane-bending vibrations of the carbonyl (C=O) group, respectively. A characteristic band on the spectra of the thioester networks are stretching vibrations of the carbonyl group, which in this case occur at 1724–1725 cm^−1^. In addition, for thioester-containing polymers (especially those with 20% thioester addition), the appearance of stretching vibration from the –C(=O)–S group can be seen at 1678–1685 cm^−1^. Another important signal is the C-S stretching vibration derived from the –C(=O)–S group shown in the range of 789–794 cm^−1^. This band is the strongest in the case of 20% addition of 1,5-NAF-S-Met (or 1,4(1,5)-NAF-CH_2_S-Met), but also appears for MMA copolymers containing 5% of these thioesters.

**Figure 5:**
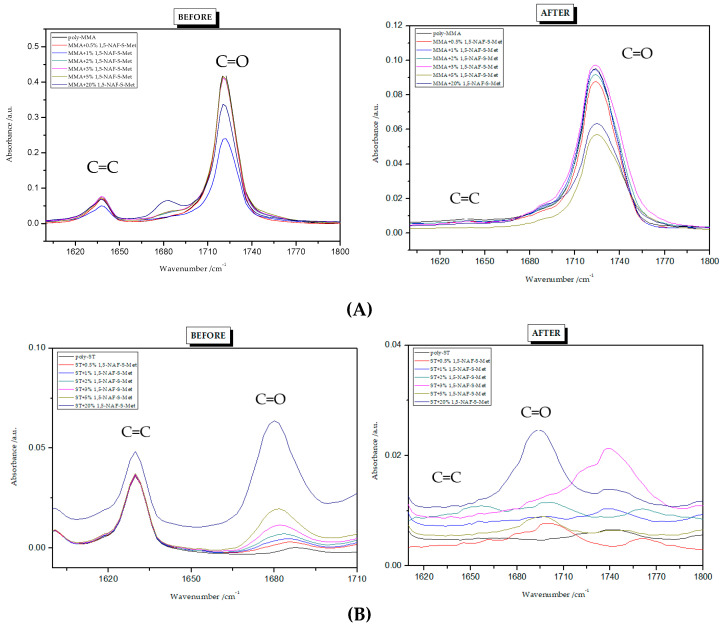
FTIR spectra of 1,5-NAF-S-Met polymers: (**A**) copolymers with MMA; (**B**) copolymers with ST.

The ATR/FT-IR spectrum (Figure 4) for ST copolymers containing 5% and 20% of 1,5-NAF-S-Met (or 1,4(1,5)-NAF-CH_2_S-Met) shows the appearance of vibration at 1673–1697 cm^−1^, which is attributed to the carbonyl group (C=O) present in thioester molecules. As in the previous spectrum, we are dealing with vibration at approximately 790 cm^−1^ (for polymers with 5% and 20% content of 1,5-NAF-S-Met and 1,4(1,5)-NAF-CH_2_S-Met). The presence of both these bands on the copolymer spectra indicates a well-carried out polymerization process. Additionally, in the spectra of ST copolymers (Figure 5), C–H stretching vibrations of methyl groups at 2849–2922 cm^−1^ and stretching vibrations of the aromatic groups C_Ar_-H at 3025–3060 cm^−1^ are observed. Other absorption bands present in the spectra of ST copolymers are Ar-H deformation vibrations at 696–757 cm^−1^.

### 8.3. Conversion of Unsaturated Bonds

**Figure 6:**
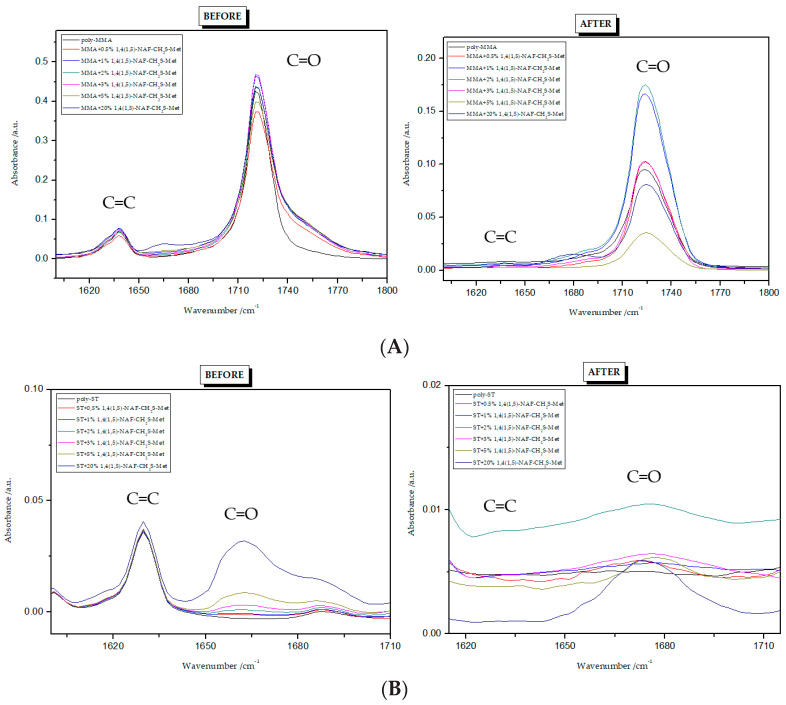
FTIR spectra of 1,4(1,5)-NAF-CH_2_S-Met polymers: (**A**) copolymers with MMA; (**B**) copolymers with ST.

To find the conversion degree, intensities of peaks responsible for stretching vibrations of C=C (1637 cm^−1^) and C=O (1720 cm^−1^) groups before and after polymerization were compared. The FTIR spectra for all polymers are presented in Figure 5 and Figure 6. The conversion degree dependence on the thioester content is shown in Table 3. The degree of crosslinking of the polymers obtained is high and ranges from 93% to 100%. Its values are higher for ST copolymers compared to MMA copolymers. Standard deviations from three measurements are given in brackets in Table 3.

### 8.4. DSC Analysis

**Figure 7:**
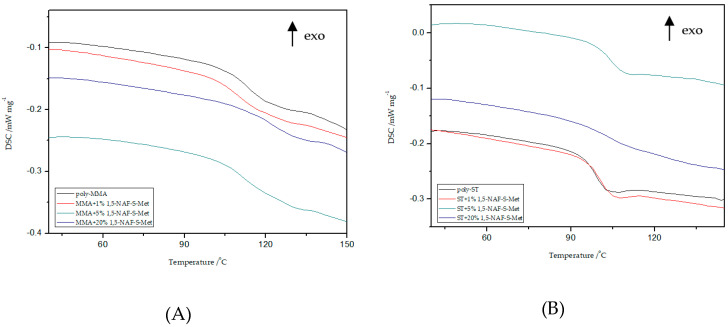
Influence of various addition of 1,5-NAF-S-Met on the glass transition temperature (T_g_) values of MMA- (**A**) or ST- based (**B**) polymers.

**Figure 8:**
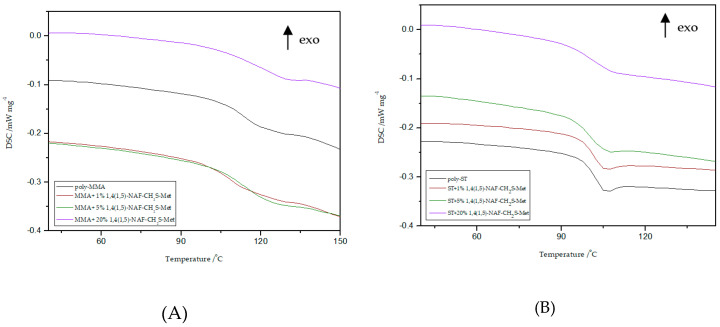
Influence of various addition of 1,4(1,5)-NAF-CH_2_S-Met on the T_g_ values of MMA- (**A**) or ST- based (**B**) polymers.

Thermal properties of all MMA and ST polymers reported in this work were investigated by DSC and TG/DTG. Polymers with addition of dithioesters (1,5-NAF-S-Met and 1,4(1,5)-NAF-CH_2_S-Met) are analyzed by DSC to characterize properties including glass transition temperature, decomposition temperature, and enthalpy of decomposition. Data and curves were acquired from the second heat endotherm so that all prepared samples could be compared without adulteration by their synthetic processing and annealing history. The results of T_g_ values of selected materials with 1%, 5%, and 20% addition of new thioesters are presented in Figure 7 and Figure 8. The glass transition temperatures of poly-ST and poly-MMA are also shown in Figure 7 and Figure 8 for the sake of comparison. The results of the DSC analysis for poly-MMA and poly-ST were published in one of our previous works [56].

Based on the presented results, it is clearly visible that the glass transition temperature for poly-ST is 101 °C and it is in good agreement with the reported values [57,58]. Comparing the obtained ST copolymers with 1,5-NAF-S-Met and with 1,4(1,5)-NAF-CH_2_S-Met vs. poly-ST, nonsignificant differences in T_g_ values can be seen. The T_g_ values for 1,5-NAF-S-Met-based ST ranged from 100 °C to 103 °C, and for 1,4(1,5)-NAF-CH_2_S-Met-based ones between 95 and 102 °C. In addition, as can be seen in Table S3, the decomposition temperatures for all synthesized ST copolymers are in the range of 380–418 °C. It should be noted that with the increase of thioester addition in the structure of ST copolymers, their thermal resistance decreases. The largest decrease in degradation temperature was noted for the 20% addition of 1,5-NAF-S-Met (T_d_ = 380 °C) compared to poly-ST (T_d_ = 418 °C).

It can be also observed that with increasing the number of added thioesters (to MMA copolymers) from 0.5% to 5%, the T_g_ values of obtained materials lightly decrease. However, when the number of thioesters is higher than 5%, the changes in T_g_ values become more significant and these values increase. The T_g_ values are 122 °C for the MMA + 20% 1,5-NAF-S-Met and 120 °C for the MMA + 20% 1,4(1,5)-NAF-CH_2_S-Met. The two-stage decomposition is observed for MMA copolymers, especially in the case of addition of 1,5-NAF-S-Met. The first peak (in a two-stage decomposition) occurs in the temperature range 261–358 °C, while the second between 358 and 398°C. The degradation temperatures of MMA copolymers with addition of aromatic thioesters were higher compared to poly-MMA. The increase of T_d_ with the increasing weight fraction of 1,5-NAF-S-Met (or 1,4(1,5)-NAF-CH_2_S-Met) was indicated. The complete characteristics of all synthesized MMA and ST polymers can be found in Supplementary Materials (Figures S4, S5 and Tables S3, S4).

### 8.5. TG/DTG Analysis

**Figure 9:**
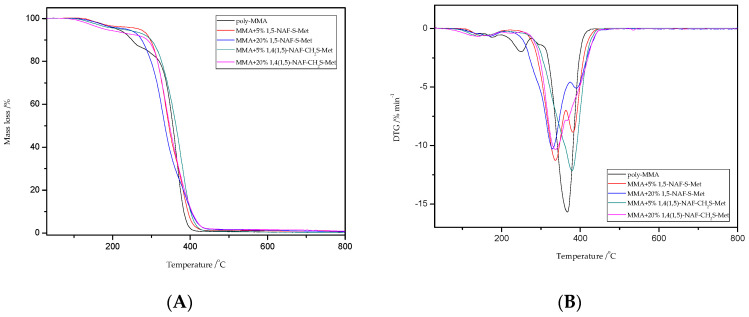
The TG (**A**) and DTG (**B**) curves of poly-MMA and its copolymers with 5% and 20% content of 1,5-NAF-S-Met and 1,4(1,5)-NAF-CH_2_S-Met.

**Figure 10:**
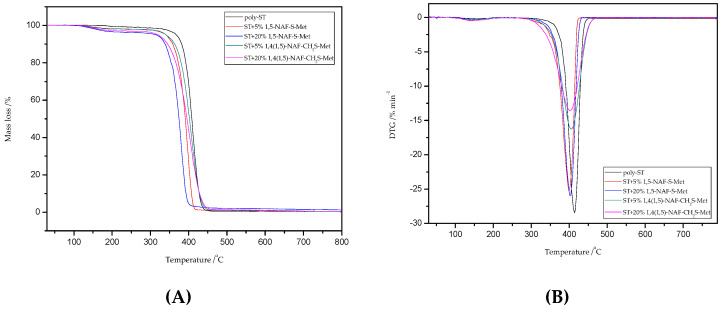
The TG (**A**) and DTG (**B**) curves of poly-ST and its copolymers with 5% and 20% content of 1,5-NAF-S-Met and 1,4(1,5)-NAF-CH_2_S-Met.

Thermal stability properties of the synthesized thioester-containing polymers were investigated by thermogravimetric Analysis (Figure 9 and Figure 10, and Table 4). The polymer samples were heated from room temperature to 800 °C at a rate of 10 °C min^−1^ with a helium purging rate of 20 mL min^−1^. Polymer decomposition temperatures reported here are the temperatures of 2%, 5%, 10%, and 50% weight loss (Table 4), respectively. After an initial weight loss at T_2_ associated with the residual solvents and humidity absorbed into samples from the environment, the main weight loss starts at about 250 °C for the thioester-containing polymers (based on MMA, Figure 9), and at about 310 °C for the thioester-containing polymers (based on ST, Figure 10). It should be noted that when the degradation temperatures at their 10% weight loss are compared, the T_10_ values of MMA copolymers were also lower than those of similar thioester-containing polymers based on ST. However, the addition of thioesters (1,5-NAF-S-Met or 1,4(1,5)-NAF-CH_2_S-Met) to methyl methacrylate increases the thermal stability of the resulting polymers by up to 20 °C. The thioester-containing polymers based on MMA exhibit lower thermal stability.

TG/DTG curves for poly-MMA show three stages of mass loss in the thermal degradation at T_max_ = 180; 250, and 367 °C; which is consistent with the literature reports [59,60,61]. In addition, the MMA copolymers presented typical two-stage decomposition profiles as shown in Figure 9 according to their TG/DTG curves. The two peaks may refer to two different components in the polymer structure that have different decomposition temperature. Comparing the peak areas of the two decomposition components, one can observe a trend of increasing peak area of the first component (<350 °C) with the increase of percentage content of obtained thioesters. It is likely that thioester moieties have been preferentially cleaved at 300–350 °C to release volatile side-group fragments, and that the polymer backbone is decomposed at about 400 °C.

Thermal degradation of poly-ST is documented and occurs by the chain scission and free radical diffusion mechanism [62]. In the case of ST copolymers, we can see one stage decomposition in the range of 300–470 °C with maximum temperatures of 387–414 °C (Figure 10). Additionally, the 1,4(1,5)-NAF-CH_2_S-Met results in better thermal stability of ST copolymers than those with 1,5-NAF-S-Met. A large solid residue at 800 °C (R) is observed for the MMA + 20% 1,4(1,5)-NAF-CH_2_S-Met copolymer (1.52% residue), and for all other samples it has been found to be between 0.22% and 1.26%, including poly-MMA and poly-ST.

The thermal stability of thioester-containing polymers has been studied and published by other authors. For example, You et al. [63] synthesized a new poly(thioester)s (PTE)s containing sulfide and alicyclic units in polymer chains. They showed good thermal properties such as 5% weight loss temperature (T_5_) in the range 307–301 °C and T_g_s in the range 65–110 °C due to the introduction of alicyclic and ester groups in the polymer chains, which endow chain rigidity. Song et al. [64] prepared a functional polyethylene carrying thioester pendants via ring-opening metathesis polymerization (ROMP) of alkyl cyclopent-3-enecarbothioate catalyzed by a ruthenium-based catalyst. They investigated the thermal properties of obtained materials and they reported that the thioester groups were decomposed at 300–400 °C to release volatile fragments of the side groups, and decomposition of polymer backbone was initiated at temperature above 400 °C. Similar conclusions were reported by the research of Aksakal et al. [65]. In summary, TGA analysis indicated that all the thioester-containing polymers exhibited moderate thermal stability.

### 8.6. Optical Properties

Polymers containing specific chromophores in their structure, such as aromatic groups, conjugated multiple bonds or UV-absorbing atoms (e.g., a sulfur atom) can be examined by UV/vis spectroscopy. Among other things, the refractive index values depend on the content of sulfur atoms in the polymer [66,67]. The production of optical materials such as lenses, prisms, optical waveguides, and disks substrates requires the use of colorless and transparent materials. Different properties of transparent synthetic resins are required as optical materials, among which the refractive index is quite important [68,69]. For example, Matsuda et al. [69] prepared transparent resins which have high refractive index by using good copolymerizable crosslinking thiomethacrylates with other vinyl monomers.

**Figure 11:**
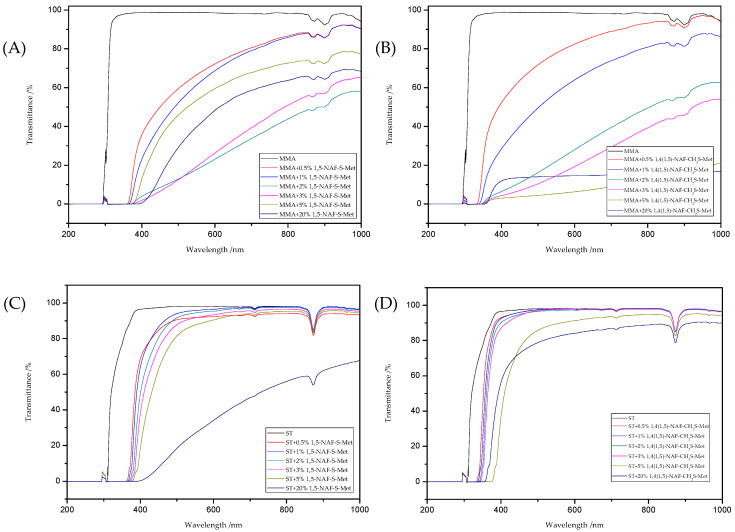
The UV/vis transmittance spectra of the prepared liquid compositions with the different addition of dithioesters: MMA+1,5-NAF-S-Met (**A**), MMA+1,4(1,5)-NAF-CH_2_S-Met (**B**), ST+1,5-NAF-S-Met (**C**), and ST+1,4(1,5)-NAF-CH_2_S-Met (**D**).

The refractive indexes and transmittance data obtained for all liquid compositions were presented in Table 5. The Figure 11provides the UV/vis spectra of those compositions. As a result of using thioesters as additives to polymers based on MMA or ST, an increase in refractive indexes was observed (Table 5). As the amount of 1,5-NAF-S-Met (or 1,4(1,5)-NAF-CH_2_S-Met) increases, the refractive index also increases. UV/vis absorption studies have shown a decrease in the transparency of the prepared liquid compositions. ST-based materials exhibit a better transparency compared to the same MMA-based products.

### 8.7. Hardness Tests

**Figure 12:**
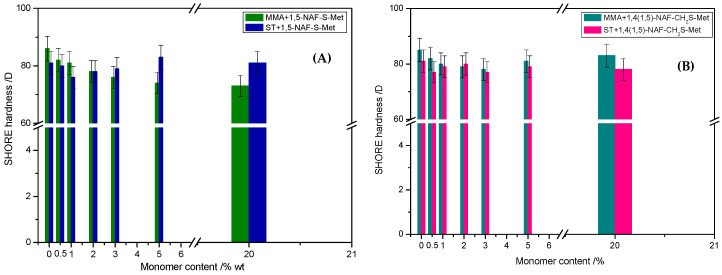
The values of Shore hardness tests of obtained cross-linked polymers with addition of 1,5-NAF-S-Met (**A**) and 1,4(1,5)-NAF-CH_2_S-Met (**B**).

The mechanical properties of thioester-containing polymers are determined by analysis of hardness tests. As can be seen from the data presented in Figure 12, the 1,5-NAF-S-Met-based MMA showed lower hardness (73–82 vs. 78–83 °Sh in Scale D) than the analogous MMA copolymers with 1,4(1,5)-NAF-CH_2_S-Met. In the case of ST copolymers, inverse correlation was observed. The ST+1,5-NAF-S-Met copolymers exhibit higher hardness (76–83 vs. 77–80 °Sh in Scale D) compared to ST+1,4(1,5)-NAF-CH_2_S-Met copolymers. However, the values of hardness as comparable to poly-MMA and poly-ST, respectively. The obtained materials possess lower Shore hardness D values in comparison to commercially available polymethyl methacrylate (PMMA) (87–88 °Sh), polycarbonate (82–85 °Sh), and polystyrene (80 °Sh) [70].

### 8.8. The Swelling Studies of Cross-Linked Polymers

**Figure 13:**
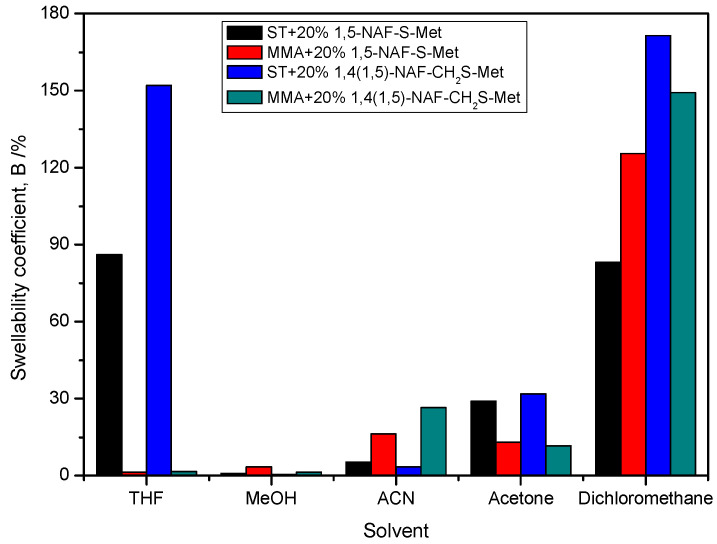
The swelling coefficients of obtained MMA and ST polymers with 20% addition of1,5-NAF-S-Met and 1,4(1,5)-NAF-CH_2_S-Met.

The procedure for measuring the swelling coefficients (B) consisted of weighing a specified mass of the polymer (about 0.2 g) and then immersing the tested polymer in selected solvents. The following solvents for swelling tests were chosen THF, MeOH, ACN, acetone, DCM, and distilled water. The results of swelling studies of the polymer materials are presented in Figure 13 and Table 6. The lowest swellability coefficients were obtained for the MMA copolymer with 20% addition of 1,5-NAF-S-Met. The ST copolymers with 20% addition of dithioesters had a higher swellability coefficient than analogous copolymers with MMA. None of the thioester-containing polymers swelled in distilled water. The highest swelling coefficients were obtained in THF and DCM.

Table 6 summarizes the swelling coefficients of the tested copolymers after 24 h, as well as after removing them from organic solvents and keeping the samples in air at various time intervals (after 15 min, 2 h, and 24 h). Based on the results obtained, it can be concluded that the polymers tested do not dissolve in these organic solvents, but only swell. After 24 h in the atmosphere of air, the polymer samples have swelling coefficients in the range of 0-16.6%, which in most cases corresponds to the values before the swelling measurements. Immersion of cross-linked polymers in solvents does not lead to their dissolution due to their chemically bonded hydrocarbon chains. However, these linkages do not prevent the swelling of cross-linked polymers.

### 8.9. Thiol-Thioester Exchange and Thiol-Methacrylate Modification Reactions

#### 8.9.1. ATR/FT-IR Analysis

**Figure 14:**
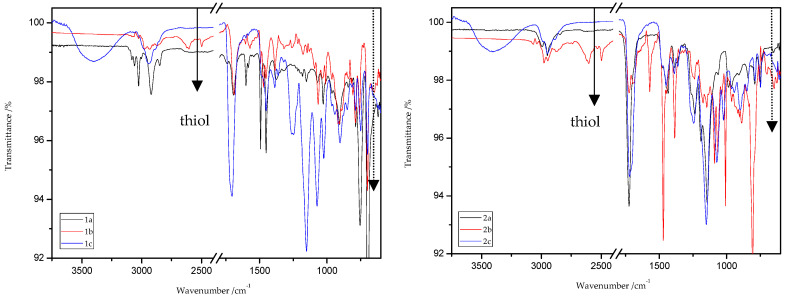
ATR/FT-IR spectra of ST (1a–c) and MMA (2a–c) copolymers.

**Figure 15:**
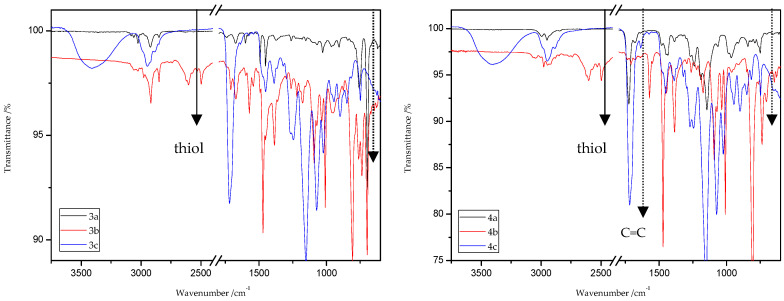
ATR/FT-IR spectra of ST (3a–c) and MMA (4a–c) copolymers.

ATR/FT-IR analyses of all obtained products and respective reagents were conducted. Figure 14 and Figure 15 show the ATR/FT-IR spectra of products obtained from MMA and ST copolymers with 20% addition of 1,5-NAF-S-Met (or 1,4(1,5)-NAF-CH_2_S-Met).

**Figure 16:**
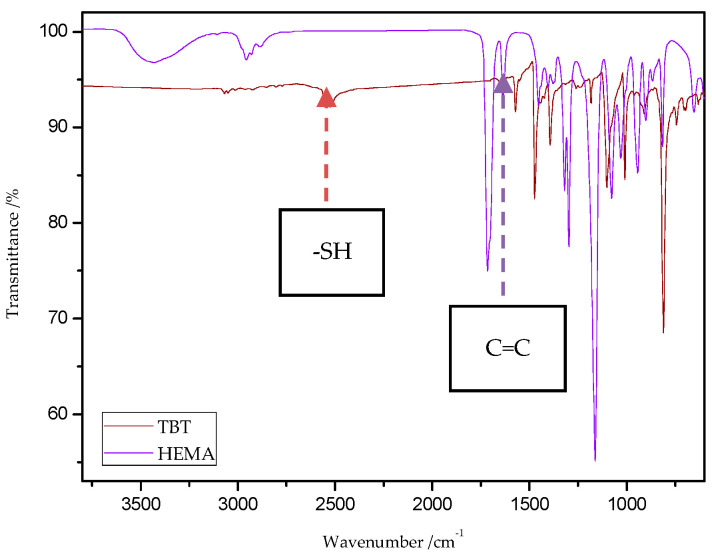
ATR/FT-IR spectra of 4,4′-thiobisbenzenethiol (TBT) and 2-hydroxyethyl methacrylate (HEMA).

The characteristic band that indicates the presence of C-S bonds is positioned in the range of 653–698 cm^−1^. If the click reaction occurred, one might expect the relative increase of this peak at the end of reaction (in IR spectra of 1c, 2c, 3c, and 4c). As indicated by the dotted lines in Figure 14 and Figure 15, a peak increase is slightly observed. ATR/FT-IR spectra of compounds obtained after thiol-thioester exchange (1b, 2b, 3b, and 4b) possess characteristic signals derived from thiol moiety in the range of 2515–2550 cm^−1^, carbonyl valency bond at 1673–1721 cm^−1^, and also the strong bands at 1570–1602 cm^−1^ which are assigned to C=C aromatic bonds. The thiol groups always converted fully, whereas the C=C stretch band indicated varying levels of conversion depending on the mixture composition. Figure 16 shows representative ATR/FT-IR spectra for TBT and 2-hydroxyethyl methacrylate (HEMA). The peaks ascribed to both the C=C double bond (1614 cm^−1^, IR spectra of HEMA) and S–H bond (at about 2554 cm^−1^, IR spectra of TBT) decreased after thiol-ene reactions.

**Figure 17:**
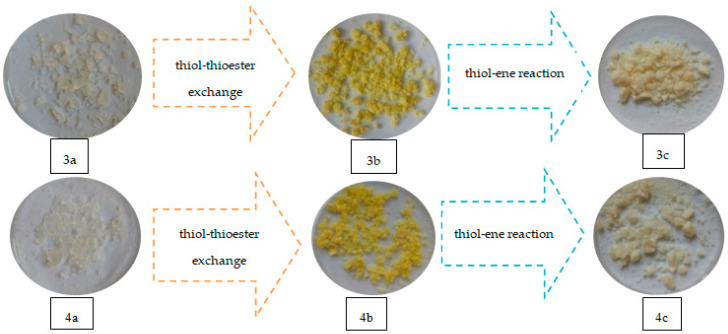
Images of materials obtained with ST (3a–c) and MMA (4a–c) copolymers with 1,4(1,5)-NAF-CH_2_S-Met.

Replacement of the thioester substituent by thiol groups (derived from TBT) resulted in a change of properties of the crosslinked polymers (compared to the original polymers with 20% addition of 1,5-NAF-S-Met or 1,4(1,5)-NAF-CH_2_S-Met), including transmutations in their physical properties. As a result of the reaction with HEMA (thiol-ene reaction), there are also noticeable physical changes in the newly obtained materials as shown in Figure 17.

#### 8.9.2. Studies of Hardness and Swelling of Materials After Thiol-Ene Reactions

Shore hardness tests show that because of the reaction of thiol-ene with 2-hydroxyethyl methacrylate, materials with lower hardness as compared to the starting materials were obtained. The modified materials possess the Shore hardness values (°Sh, in scale D), 1c—50, 2c—27, 3c—42, and 4c—20, respectively. The process of postmodification caused a decrease in hardness of about 31–63 °Sh. These products are brittle.

The swelling studies of products obtained after thiol-ene reaction were also performed. The swellability coefficients in THF, MeOH, acetone, ACN, DCM, and distilled water were studied. The results are summarized in Table 7. Based on the results presented below, one can conclude that the highest values of swelling coefficients were obtained for methanol and distilled water. One can observe that the highest swellability coefficient possesses the material marked as 2c, whereas the material designed as 3c swells to a lesser extent. In comparison to the starting copolymers based on St and MMA, we can observe that the new thiol-ene materials swell better in polar solvents.

#### 8.9.3. DSC Study

**Figure 18:**
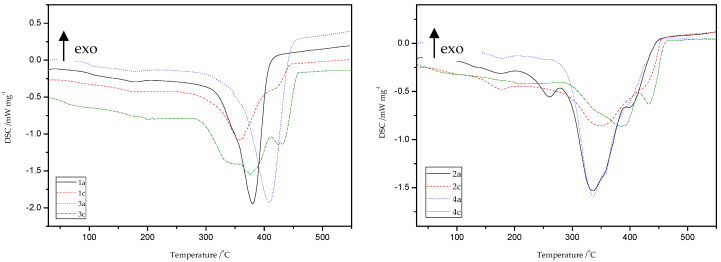
Differential scanning calorimetry (DSC) curves of starting (1a, 2a, 3a, 4a) and new (1c, 2c, 3c, 4c) materials obtained after thiol-ene reactions with HEMA.

Thermal characterizations of all obtained materials because of thiol-thioester exchanges and then thiol-ene reactions were carried out by the DSC method (Figure 18 and Table 8). The peak appears in the temperature range of 174–185 °C is related to the presence of moisture and unreacted monomers.

Generally, DSC curves of all studied materials show one, asymmetric, non-well separated endothermic signal with one, two, or three maxima (T_d_), which is directly connected with the decomposition of the studied materials. It is likely that more than one phase exists in the samples of obtained polymers, which can be deduced from the presence of double peaks on the DSC curves. This may be due to the occurrence of a mixture of ST (1c, 3c) and MMA (2c, 4c) copolymers, respectively, as well as HEMA homopolymer in the structure of the polymers obtained. Products obtained because of depolymerization of the network and subsequent re-crosslinking (1b–4b and 1c–4c) have different thermal properties compared to the original materials (1a–4a). In the case of ST materials, the thiol-ene reaction with HEMA caused a slight decrease in the value of decomposition temperatures, as well as the appearance of an endothermic peak at about 413–434 °C. This peak is associated with the decomposition of the aromatic part present in the polymer chain. The main decomposition of ST copolymers occurs in the temperature range of 280–450 °C with the maximum peak at 347–413 °C. The DSC thermogram of the polymers gave a T_g_ value at 83–112 °C. In consequence of the transformations performed, the changes were also noted on DSC curves for thioester-containing polymers based on MMA. These materials are characterized by presence of endothermic peak in the range of 336 to 441 °C which is attributed to main decomposition of polymer units, such as aliphatic and carbonyl-containing chains. Therefore, new polymers (2c, 4c) exhibit higher decomposition temperatures compared to the starting materials (2a, 4a) of more than 20 °C.

To sum up, the reprocessing of starting materials resulted in reduction of the glass transition temperature of the final products and caused changes in thermal behavior of new materials.

## 9. Conclusions

Synthesis, structure, and polymerization two aromatic dithioesters based on naphthalene are reported. The presented results confirmed that the addition of thioesters (1,5-NAF-S-Met or 1,4(1,5)-NAF-CH_2_S-Met) to methyl methacrylate or styrene copolymers allowed preparation of crosslinked materials with good thermal stability. The double bond conversion of the polymers reached high levels of 93–100%. Overall, the thioester-containing polymers based on MMA exhibit more decreased thermal stability than those with ST, addition from 0.5% to 5% of thioester results in a decrease of the T_g_ values. The prepared liquid compositions also possess good optical properties, especially those based on ST. The addition of dithioesters results in an increase in the refractive indexes values. In the case of using a 20% addition of 1,5-NAF-S-Met or 1,4(1,5)-NAF-CH_2_S-Met to ST compositions, the refractive index increased to 1558 and 1554, respectively. In general, the presence of sulfur atom in the structure of copolymers decreased the hardness values of these materials, which is most noticeable for MMA-based polymers with different addition of 1,5-NAF-S-Met. The presence of a thioester moiety in the polymer allowed their reused by application of thiol-thioester exchange modification and depolymerization reactions. For this purpose, thiol-ene modification with HEMA was successfully performed. As a result, T_g_ has decreased of about 20 °C for ST copolymers, and about 60 °C for MMA copolymers, respectively. Through the thiol-ene reactions, reprocessing was carried out to obtain softer and more heat-resistant materials. Thiol-thioester exchange reactions and thiol-ene reactions are effective methods for modification of polyesters. Products obtained via these techniques are easily processable and recyclable. Thiol-ene reactions can be useful in surface functionalization, re-use of crosslinking materials, and polymerization of renewable monomers in synthesis of sulfur-containing polymers. Additionally, the obtained thioesters may find application as additives to polymers used in photonics (e.g., coatings in optical fiber).

## Figures and Tables

**Table 1:** The amounts of monomers used in copolymerization.

Thioester Methacrylate	Comonomer	Thioester Monomer—Comonomer Feed Ratios (w/w)	Amount of Sulfur Monomer /g	Amount of Comonomer/g	Yield of Polymerization /%	
					MMA Polymers	ST Polymers
1,5-NAF-S-Met	MMA (or ST)	-	-	2	97	99
		1:200	0.01	2	97	98
		1:100	0.02	2	96	97
		1:50	0.04	2	96	96
		1:33	0.06	2	95	97
		1:20	0.1	2	97	98
		1:5	0.4	2	97	98
1,4(1,5)-NAF-CH_2_S-Met	MMA (or ST)	-	-	2	97	99
		1:200	0.01	2	97	99
		1:100	0.02	2	95	97
		1:50	0.04	2	96	96
		1:33	0.06	2	97	98
		1:20	0.1	2	97	99
		1:5	0.4	2	95	97

**Table 2:** Simplified designation of polymer sample names.

Material	Simplified Designation of Sample
ST+1,5-NAF-S-Met	1a
ST+1,5-NAF-S-Met+TBT	1b
ST+1,5-NAF-S-Met+TBT+HEMA	1c
MMA+1,5-NAF-S-Met	2a
MMA+1,5-NAF-S-Met+TBT	2b
MMA+1,5-NAF-S-Met+TBT+HEMA	2c
ST+1,4(1,5)-NAF-CH_2_S-Met	3a
ST+1,4(1,5)-NAF-CH_2_S-Met+TBT	3b
ST+1,4(1,5)-NAF-CH_2_S-Met+TBT+HEMA	3c
MMA+1,4(1,5)-NAF-CH_2_S-Met	4a
MMA+1,4(1,5)-NAF-CH_2_S-Met+TBT	4b
MMA+1,4(1,5)-NAF-CH_2_S-Met+TBT+HEMA	4c

**Table 3:** Degree of conversion (%) of studied polymers.

% w/w 1,5-NAF-S-Met (or 1,4(1,5)-NAF-CH_2_S-Met)	Degree of Conversion (DC)/%			
	ST+1,5-NAF-S-Met	MMA+1,5-NAF-S-Met	ST+1,4(1,5)-NAF-CH_2_S-Met	MMA+1,4(1,5)-NAF-CH_2_S-Met
0	100 (-)	95 (3)	100 (-)	95 (3)
0.5	100 (-)	97 (2)	100 (-)	96 (2)
1	100 (2)	95 (3)	99 (1)	95 (2)
2	99 (2)	96 (2)	99 (1)	95 (3)
3	99 (1)	96 (2)	99 (2)	94 (3)
5	99 (2)	98 (2)	99 (2)	93 (2)
20	98 (2)	97 (3)	98 (2)	93 (2)

**Table 4:** Thermal properties of poly-MMA, poly-ST, and obtained poly(thiomethacrylate)’s.

Copolymer	Temperature/ °C						R/%
	*^a^ T_2_*	*^b^ T_5_*	*^c^ T_10_*	*^d^ T_50_*	*^e^ T_f_*	*^f^ T_max_*	
poly-MMA	155	240	272	355	435	180; 250; 367	0.40
MMA + 5% 1,5-NAF-S-Met	162	270	298	345	436	157; 337; 381	0.69
MMA + 20% 1,5-NAF-S-Met	161	236	275	335	450	171; 328; 391	1.17
MMA + 5% 1,4(1,5)-NAF-CH_2_S-Met	148	228	285	345	445	149; 346	0.90
MMA + 20% 1,4(1,5)-NAF-CH_2_S-Met	131	181	290	347	460	142; 337; 367	1.52
poly-ST	329	368	384	409	460	414	0.26
ST + 5% 1,5-NAF-S-Met	262	341	359	391	440	144; 397	0.60
ST + 20% 1,5-NAF-S-Met	158	314	338	375	434	139; 387	1.26
ST + 5% 1,4(1,5)-NAF-CH_2_S-Met	253	341	364	402	470	141; 405	0.22
ST + 20% 1,4(1,5)-NAF-CH_2_S-Met	168	321	345	396	470	139; 402	0.36

^a^ temperature corresponding to 2% weight loss; ^b^ temperature corresponding to 5% weight loss; ^c^ temperature corresponding to 10% weight loss; ^d^ temperature corresponding to 50% weight loss; ^e^ temperature corresponding to the final decomposition; and ^f^ the maxima peak temperature.

**Table 5:** Optical properties.

% Thioester	Refractive Index			
	ST+1,5-NAF-S-Met	MMA+1,5-NAF-S-Met	ST+1,4(1,5)-NAF-CH_2_S-Met	MMA+1,4(1,5)-NAF-CH_2_S-Met
*0*	1.5440	1.4142	1.5440	1.4142
*0.5*	1.5448	1.4143	1.5442	1.4142
*1*	1.5452	1.4146	1.5445	1.4152
*2*	1.5460	1.4153	1.5452	1.4166
*3*	1.5470	1.4190	1.5458	1.4196
*5*	1.5485	1.4221	1.5470	1.4238
*20*	1.5580	1.4473	1.5540	1.4402

**Table 6:** Swelling studies.

Solvent	Swelling Coefficients, B /%			
	After 24 h in Solvent	After 15 Min in Air	After 2 h in Air	After 24 h in Air
**ST+20% 1,5-NAF-S-Met**				
*THF*	86.1	38.8	23.5	11.0
*MeOH*	0.9	0.2	0.1	0.05
*ACN*	5.3	4.2	3.0	1.7
*Acetone*	29.0	22.8	17.3	8.9
*DCM*	83.0	34.1	11.0	1.2
**MMA+20% 1,5-NAF-S-Met**				
*THF*	1.4	0.1	0	0
*MeOH*	3.4	2.1	1.5	0.6
*ACN*	16.3	12.1	9.3	3.3
*Acetone*	13.0	8.7	5.8	3.2
*DCM*	125.6	43.8	18.3	11.1
**ST+20% 1,4(1,5)-NAF-CH_2_S-Met**				
*THF*	152.0	113.0	29.6	16.6
*MeOH*	0.5	0.3	0.1	0.03
*ACN*	3.4	3.1	2.5	0.9
*Acetone*	31.8	27.0	21.2	11.7
*DCM*	171.4	33.7	1.9	0.4
**MMA+20% 1,4(1,5)-NAF-CH_2_S-Met**				
*THF*	1.7	0.6	0.5	0.1
*MeOH*	1.4	0.9	0.4	0
*ACN*	26.5	20.9	15.6	7.1
*Acetone*	11.6	6.8	3.9	2.2
*DCM*	149.2	30.0	7.5	1.1

**Table 7:** Swelling properties.

Solvent	Sample			
	1c	2c	3c	4c
	Swellability Coefficient, B/%			
THF	19	43	25	70
MeOH	205	352	114	179
ACN	14	45	17	19
Acetone	44	84	41	50
DCM	25	55	16	19
Distilled Water	93	138	72	90

**Table 8:** Results of DSC analysis.

Sample	DSC Data		
	T_g_/°C	T_d_/°C	ΔH_d_/J g^−1^
1a	103	380	515
1b	112	354	294
1c	83	356; 413	406
2a	122	261; 336; 398	823
2b	135	302; 338; 353	340
2c	84	349; 422	458
3a	102	408	741
3b	-	347; 367; 472	201
3c	105	348; 376; 434	703
4a	120	337; 358	764
4b	-	338; 441	253
4c	60	384; 433	432

## Supplementary Materials

The following are available online at https://www.mdpi.com/1996-1944/13/13/3021/s1, Figure S1: Scheme of preparation of naphthalene-1,5-dithiol, Figure S2: Reaction of chloromethylation of naphthalene (A) and reaction of 1,5-bis(chloromethyl)naphthalene with thiourea (B), Figure S3: The comparison of ATR/FT-IR spectra of 1,5-NAF-SH (A) with 1,5-NAF-S-Met (B) and 1,4(1,5)-NAF-CH_2_SH (C) with 1,4(1,5)-NAF-CH_2_S-Met (D), Figure S4: The DSC curves of MMA and ST copolymers with 1,5-NAF-S-Met, Figure S5: The DSC curves of MMA and ST copolymers with 1,4(1,5)-NAF-CH_2_S-Met, Table S1: Data of ATR/FT-IR analysis of obtained MMA copolymers with addition of aromatic thioesters (1,5-NAF-S-Met and 1,4(1,5)-NAF-CH_2_S-Met), Table S2: Data of ATR/FT-IR analysis of obtained ST copolymers with addition of aromatic thioesters (1,5-NAF-S-Met and 1,4(1,5)-NAF-CH_2_S-Met), Table S3: Thermal properties of St copolymers with aromatic dithioesters, Table S4: Thermal properties of MMA copolymers with aromatic dithioesters.
