# Supplementary material for: Cross-Linked Polythiomethacrylate Esters Based on Naphthalene—Synthesis, Properties and Reprocessing
Source: Materials (Basel). 2020 Jul 6;13(13):3021. doi: 10.3390/ma13133021 (PMC7372384; doi:10.3390/ma13133021)
Supplement: Supplementary file 1 [file materials-13-03021-s001.pdf]

materials

SUPPLEMENTARY MATERIALS

Cross-Linked Polythiomethacrylate Esters Based on Naphthalene – Synthesis, Properties, and Reprocessing

Karolina Fila[1]*, Beata Podkościelna[1] and Maciej Podgórski[1,2]

[1]Department of Polymer Chemistry, Institute of Chemical Sciences, Faculty of Chemistry, Maria Curie-Skłodowska University, Maria Curie-Skłodowska Sq. 5, 20-031 Lublin, Poland; beatapod@poczta.umcs.lublin.pl (B.P.); maciej.podgorski@colorado.edu (M.P.)
[2]Department of Chemical and Biological Engineering, University of Colorado, UCB 596, Boulder, Colorado 80303, United States
*Correspondence: karolina.fila@poczta.umcs.lublin.pl

Received: date; Accepted: date; Published: date

S1. Synthesis of thiols

S1.1. Synthesis of naphthalene-1,5-dithiol (1,5-NAF-SH)

S1.1.1. Chlorosulfonation of naphthalene

To 70 g of a naphthalene melt maintained at 80°C in a 500 ml four-neck flask equipped with a mechanical stirrer, thermometer, gas discharge pipe and a dropping funnel, 150 ml of chlorosulfonic acid were added from a dropping funnel during a period of 3 h, while continuously stirring. HCl gas was evolved in the course of the resulting exothermic reaction, the bulk of which took place during the first half hour. Then the flask was placed in an ice bath and 150 ml of chlorosulfonic acid was again dropped in at 10 °C for 1 h. While warming to room temperature stirring was continued for an additional 2 h, at the end of which time precipitation took place. The precipitate was filtered and washed twice, each time with 15 ml of chlorosulfonic acid. The resulting white solid were then slurried in 200 ml of ice water, filtered, washed with a small amount of distilled water and finally dried, yielding 66 g of naphthalene-1,5-disulfonyl dichloride. The obtained precipitate was then purified by crystallization from 250 cm^3 of benzene. 50 g of a white, crystalline precipitate was obtained; yield 28%.

S1.1.2. Reduction of naphthalene-1,5-disulfonyl dichloride

To a flask with a capacity of 1000 mL equipped with a mechanical stirrer and thermometer were added 200 g of $SnCl_2*H_2O$ and 700 mL of CH_3COOH. 100 g of ammonium chloride was placed in a 2000 mL conical flask, then 150 ml of sulfuric acid were added dropwise to it. Then the gaseous HCl was passed through the mixture in a flask which was cooled to <5 ° C until a clear solution was obtained. In the next stage 25 g of naphthalene-1,5-sulfonyl chloride were added to the cooled reduction liquid prepared in this way. Stirring was continued for 2 h. The contents of the flask were then poured into a beaker containing 1500 mL of distilled water. The formed precipitate was dissolved in an aqueous NaOH solution, and then acidified with 100 mL of HCl. The obtained naphthalene-1,5-dithiol was recrystallized from a mixture of CH_3COOH and HCl. 11 g (74% yield) of a light yellow solid was obtained. **Figure S1** presents the synthesis of naphthalene-1,5-dithiol.

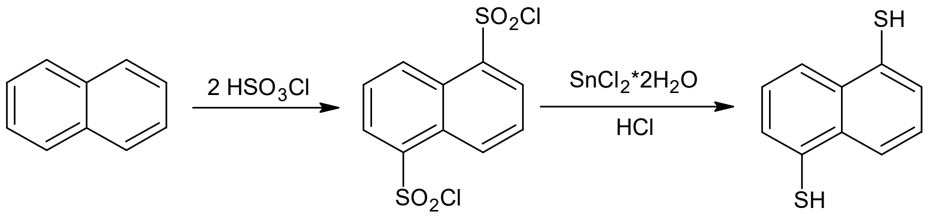

Figure S1. Scheme of preparation of naphthalene-1,5-dithiol.

S1.2. Synthesis of naphthalene-1,4(1,5)-di(ylmethanethiol) (1,4(1,5)-NAF-CH_2SH)

S1.2.1. Reaction of naphthalene with paraformaldehyde

100 g of naphthalene, 95 g of paraformaldehyde, 88 mL of glacial acetic acid, and 280 ml of concentrated hydrochloric acid were put into the 1000 mL three-necked flask filled with the reflux condenser, stirrer, and thermometer. This mixture was heated in a water bath at 80–85 °C and vigorously stirred for 6 hours. After cooling to 15–20 °C, it was transferred to a 2000 mL separatory funnel and the crude product was washed first with two 500 mL portions of distilled water, then with 500 ml of 10% sodium chloride solution, and finally with 500 mL of cold water. **Figure S2A** presents the structural formulas of the obtained products: 1,4 and 1,5-bis(chloromethyl)naphthalene isomers (1:1) and 1-chloromethylnaphthalene. The monosubstituted derivative is a liquid and as a result of filtration it is removed from the resulting mixture. The precipitate containing the mixture of isomers 1,4- and 1,5-bis(chloromethyl)naphthalene was washed with cyclohexane and then crystallization was conducted using 70 ml of ethylene chloride. After crystallization 38 g of pure solid 1,5(1,4)-bis(chloromethyl)naphthalene (yield 38%) was obtained.

S1.2.2. Reaction of 1,5(1,4)-bis(chloromethyl)naphthalene with thiourea

In the 1000 ml round bottom flask fitted with a reflux condenser, 52 g of thiourea in 400 mL of water was dissolved and then 70 g of 1,5(1,4)-bis(chloromethyl)naphthalene and 100 mL of 96% ethanol were added. The flask was heated to 90°C and kept under gentle boiling for 1.5 h. A solution of NaOH (75 g NaOH + 270 ml H_2O) was prepared, poured into the flask and heated for 1 h. After cooling the solution was filtered under the reduced pressure. 120 ml of HCl was added in portions to the filtrate. The formed precipitate was filtered off. The compound was again purified by precipitation with the solution of NaOH and HCl. 60 g of naphthalene-1,5(1,4)-di(ylmethanethiol) was obtained. Then crystallization with 240 ml of acetic acid was carried out. 40 g of recrystallized naphthalene-1,5(1,4)-di(ylmethanethiol) was obtained as a light yellow solid. **Figure S2B** shows the modification scheme for the naphthalene-1,5-di(ylmethanethiol) isomer, the reaction is analogous for the naphthalene-1,4-di(ylmethanethiol).

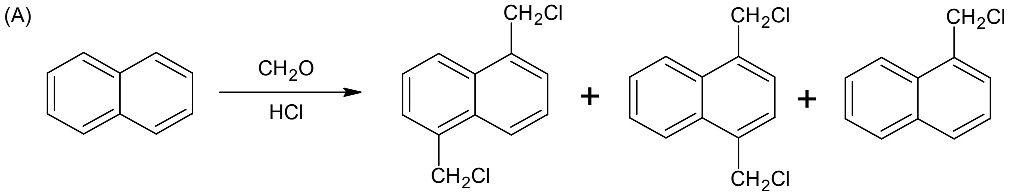

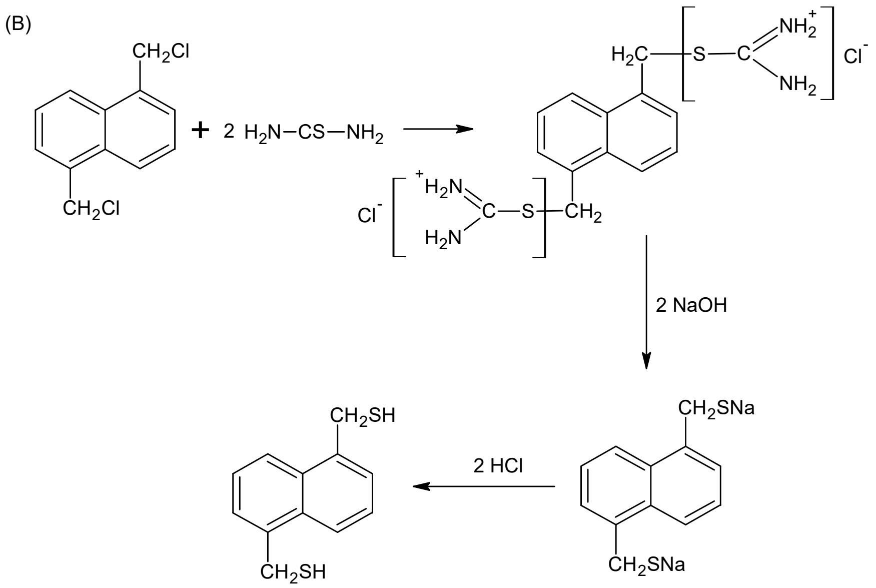

Figure S2. Reaction of chloromethylation of naphthalene (**A**) and reaction of 1,5-bis(chloromethyl)naphthalene with thiourea (**B**).

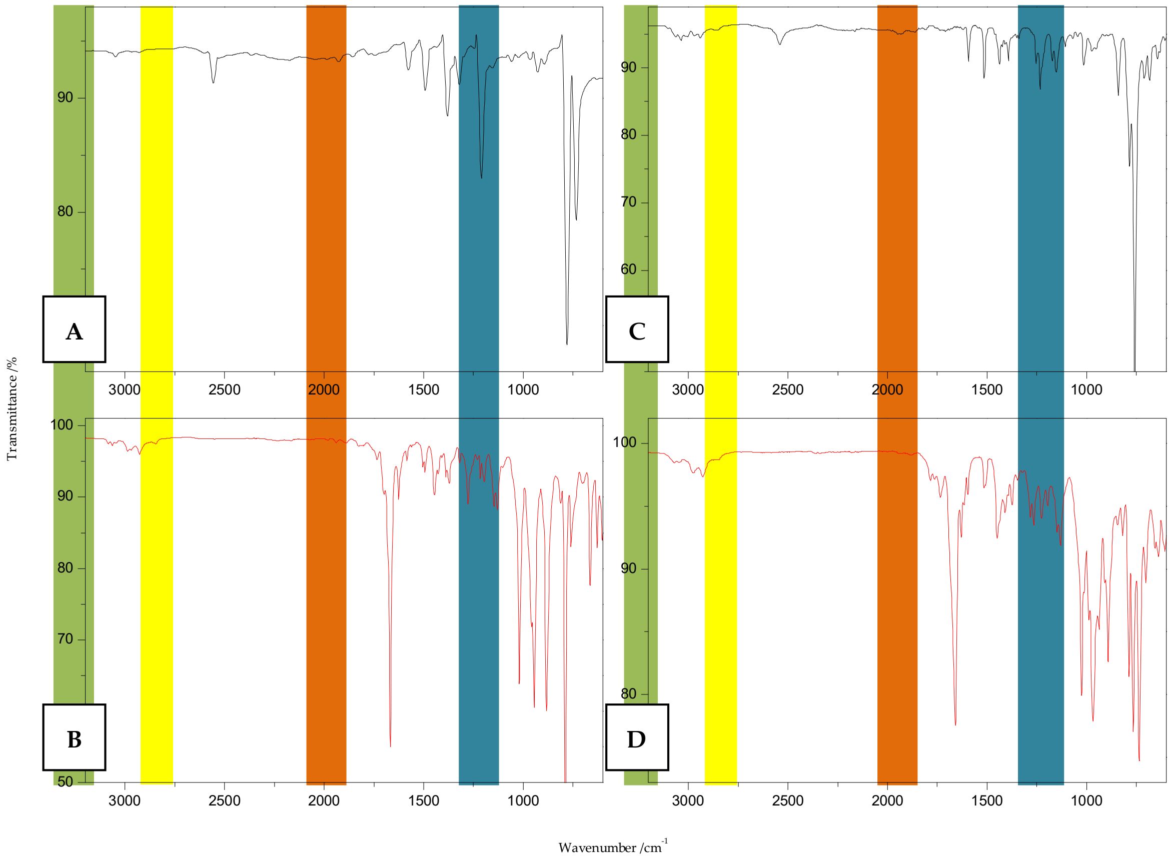

Figure S3. The comparison of ATR/FT-IR spectra of 1,5-NAF-SH (**A**) with 1,5-NAF-S-Met (**B**) and 1,4(1,5)-NAF-CH2SH (**C**) with 1,4(1,5)-NAF-CH2S-Met (**D**).

Table S1. Data of ATR/FT-IR analysis of obtained MMA copolymers with addition of aromatic thioesters (1,5-NAF-S-Met and 1,4(1,5)-NAF-CH2S-Met).

	Wavenumber /cm^{-1}							
**Polymer**	**-CH$_3$ Stretching Vibrations**	**-CH$_2$ Stretching Vibrations**	**C=O Stretching Vibrations**	**-CH$_3$ Bending Vibrations**	**-C-O-C Vibrations**	**-COS Stretching Vibrations**	**C-C Stretching Vibrations Coupled with Rocking γ(CH$_2$-) Vibration**	**C-S Stretching Vibrations**
poly-MMA	2994	2846<br>2950	1724	1435<br>1388	1144<br>1239	-	750<br>840	-
MMA+5% 1,5-NAF-S-Met	2995	2843<br>2950	1725<br>1684	1435<br>1382	1144<br>1234	794	751<br>841	653
MMA+20% 1,5-NAF-S-Met	2994	2845<br>2949	1725<br>1685	1435<br>1385	1145<br>1236	791	751<br>841	653
MMA+5% 1,4(1,5)-NAF-CH$_2$S-Met	2995	2849<br>2950	1724<br>1685	1435<br>1387	1145<br>1240	789	750<br>841	650<br>705
MMA+20% 1,4(1,5)-NAF-CH$_2$S-Met	2996	2847<br>2950	1725<br>1678	1435<br>1387	1146<br>1240	771<br>789	751<br>842	652<br>702

Table S2. Data of ATR/FT-IR analysis of obtained ST copolymers with addition of aromatic thioesters (1,5-NAF-S-Met and 1,4(1,5)-NAF-CH2S-Met).

	Wavenumber /cm^{-1}							
**Polymer**	**-C$_{Ar}$-H stretching vibrations**	**-CH$_2$ stretching vibrations**	**C=O stretching vibrations**	**C=C aromatic**	**-CH bending vibrations**	**-COS stretching vibrations**	**-C$_{Ar}$-H bending vibrations**	**-C-S stretching vibrations**
poly-ST	3026<br>3060	2849<br>2920	-	1601<br>1583	1452<br>1493	-	696<br>753	-
ST+5% 1,5-NAF-S-Met	3025<br>3060	2849<br>2921	1697	1601<br>1583	1452<br>1493	788	696<br>754	672
ST+20% 1,5-NAF-S-Met	3025<br>3059	2849<br>2921	1694	1601<br>1584	1452<br>1493	787	696<br>755	674
ST+5% 1,4(1,5)-NAF-CH$_2$S-Met	3025<br>3060	2850<br>2922	1673	1601<br>1583	1452<br>1493	789	696<br>755	665
ST+20% 1,4(1,5)-NAF-CH$_2$S-Met	3026<br>3060	2850<br>2922	1674	1601<br>1583	1452<br>1493	787	696<br>757	669

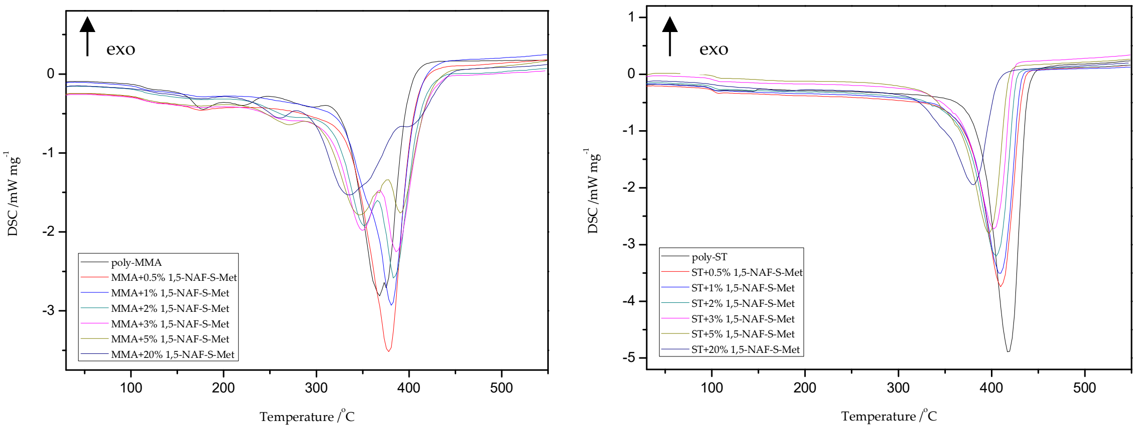

Figure S4. The DSC curves of MMA and ST copolymers with 1,5-NAF-S-Met.

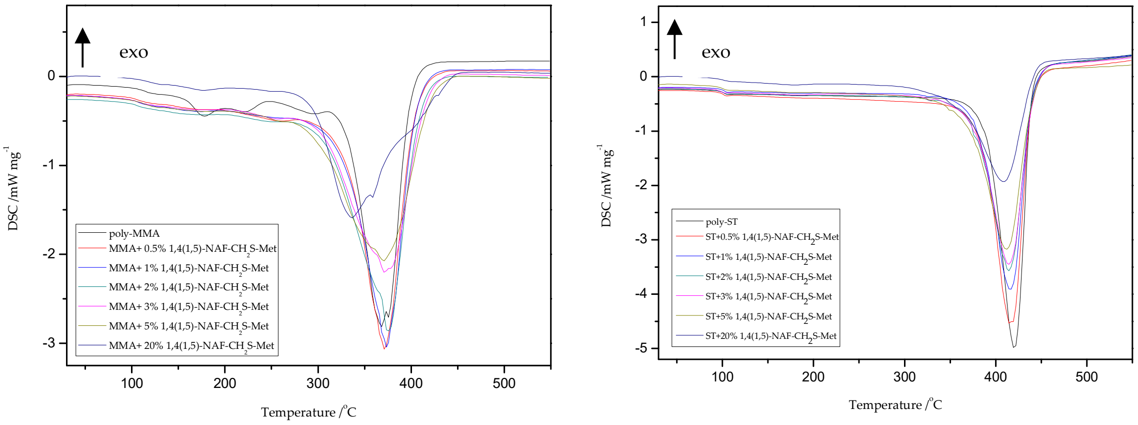

Figure S5. The DSC curves of MMA and ST copolymers with 1,4(1,5)-NAF-CH_2S-Met.

Table S3. Thermal properties of St copolymers with aromatic dithioesters.

Polymer	T_g /°C	T_{onset} /°C	T_d /°C	T_{offset} /°C	ΔH_d /J g^{-1}
poly-ST	101	350	418	455	964
ST+0.5% 1,5-NAF-S-Met	102	325	410	450	882
ST+1% 1,5-NAF-S-Met	101	320	409	440	793
ST+2% 1,5-NAF-S-Met	100	315	404	430	778
ST+3% 1,5-NAF-S-Met	102	312	403	427	694
ST+5% 1,5-NAF-S-Met	103	300	397	425	714
ST+20% 1,5-NAF-S-Met	103	280	380	420	515
ST+0.5% 1,4(1,5)-NAF-CH2S-Met	95	340	418	460	953
ST+1% 1,4(1,5)-NAF-CH2S-Met	97	335	414	462	979
ST+2% 1,4(1,5)-NAF-CH2S-Met	96	325	413	464	972
ST+3% 1,4(1,5)-NAF-CH2S-Met	99	320	412	465	928
ST+5% 1,4(1,5)-NAF-CH2S-Met	101	310	411	466	938
ST+20% 1,4(1,5)-NAF-CH2S-Met	102	300	408	465	741

Table S4. Thermal properties of MMA copolymers with aromatic dithioesters.

Polymer	T_g /°C	T_{onset} /°C	T_d /°C	T_{offset} /°C	ΔH_d /J g^{-1}
poly-MMA	114	150	178; 232; 369	430	693
MMA+0.5% 1,5-NAF-S-Met	113	276	379	432	838
MMA+1% 1,5-NAF-S-Met	110	250	381	430	743
MMA+2% 1,5-NAF-S-Met	109	230	352; 384	440	779
MMA+3% 1,5-NAF-S-Met	113	220	350; 386	450	801
MMA+5% 1,5-NAF-S-Met	111	210	271; 347; 391	450	729

MMA+20% 1,5-NAF-S-Met	122	205	261; 336; 398	446	823
MMA+0.5% 1,4(1,5)-NAF-CH$_2$S-Met	108	280	371	445	851
MMA+1% 1,4(1,5)-NAF-CH$_2$S-Met	109	278	373	442	891
MMA+2% 1,4(1,5)-NAF-CH$_2$S-Met	106	270	375	440	927
MMA+3% 1,4(1,5)-NAF-CH$_2$S-Met	106	260	371	455	987
MMA+5% 1,4(1,5)-NAF-CH$_2$S-Met	114	270	358; 371	458	995
MMA+20% 1,4(1,5)-NAF-CH$_2$S-Met	120	270	337; 358	460	764

© 2020 by the authors. Submitted for possible open access publication under the terms and conditions of the Creative Commons Attribution (CC BY) license (http://creativecommons.org/licenses/by/4.0/).
